# The RAL Enigma: Distinct Roles of RALA and RALB in Cancer

**DOI:** 10.3390/cells11101645

**Published:** 2022-05-14

**Authors:** Dillon S. Richardson, Jonathan M. Spehar, David T. Han, Prathik A. Chakravarthy, Steven T. Sizemore

**Affiliations:** The Ohio State University Comprehensive Cancer Center—James Cancer Hospital and Solove Research Institute, Department of Radiation Oncology, The Ohio State University, Columbus, OH 43210, USA; dillon.richardson@osumc.edu (D.S.R.); jonathan.spehar@osumc.edu (J.M.S.); david.han@osumc.edu (D.T.H.); chakravarthy.17@buckeyemail.osu.edu (P.A.C.)

**Keywords:** RALA, RALB, RAL, GTPases, cancer, RAS, RALBP1, endocytosis, exocytosis

## Abstract

RALA and RALB are highly homologous small G proteins belonging to the RAS superfamily. Like other small GTPases, the RALs are molecular switches that can be toggled between inactive GDP-bound and active GTP-bound states to regulate diverse and critical cellular functions such as vesicle trafficking, filopodia formation, mitochondrial fission, and cytokinesis. The RAL paralogs are activated and inactivated by a shared set of guanine nucleotide exchange factors (GEFs) and GTPase-activating proteins (GAPs) and utilize similar sets of downstream effectors. In addition to their important roles in normal cell biology, the RALs are known to be critical mediators of cancer cell survival, invasion, migration, and metastasis. However, despite their substantial similarities, the RALs often display striking functional disparities in cancer. RALA and RALB can have redundant, unique, or even antagonistic functions depending on cancer type. The molecular basis for these discrepancies remains an important unanswered question in the field of cancer biology. In this review we examine the functions of the RAL paralogs in normal cellular physiology and cancer biology with special consideration provided to situations where the roles of RALA and RALB are non-redundant.

## 1. Introduction

The RAS proteins were discovered in the 1960s and later identified as the first human proto-oncogenes [1,2,3]. HRAS, NRAS, KRAS, and more than 160 related GTPases comprise a large family of small G proteins [4]. Aside from RAS, the best studied members of this superfamily include RHO, RAB, RAN, and ARF. These small G proteins are critical mediators of numerous cellular functions, switching between active, GTP-bound, and inactive, GDP-bound forms to serve as molecular switches during intracellular signaling events [5]. The RAS superfamily influences normal cellular biology by altering signal transduction, vesicle trafficking, cytoskeletal dynamics, and nuclear import and export of cargo [6].

In addition to their important roles in normal physiology, RAS family members are frequently drivers of cancer initiation and progression. These small G proteins can be hijacked to promote cancer cell survival, proliferation, migration, invasion, and metastasis. For example, oncogenic RAS promotes cancer cell survival through its key effectors—phosphoinositide 3-kinase (PI3K) and RAF—increases proliferation through activation of FOS, JUN and ATF2, and supports oncogenic metabolism through upregulation of HIF1α [7,8,9,10,11]. Other small G proteins of the RHO family increase cancer proliferation by downregulating cell cycle inhibitors, increase tumor vasculature by increasing VEGFR2 expression, and are critical mediators of cell motility [12]. In addition to other mechanisms, overactivation of RAC1 or CDC42 promotes cancer migration and invasion by increased actin polymerization to elicit cellular polarization and secretion of matrix metalloproteases (MMPs) [13].

The RAS-like oncoproteins A and B (RALA and RALB) are small G proteins which are critical downstream of effectors of RAS, along with RAF and PI3K [14]. RAS directly activates several RAL guanine nucleotide exchange factors (RALGEFs) to encourage RALA and RALB activity. RALA, originally designated as RAL, was discovered by Pierre Chardin and colleagues through its homology to the RAS [15]. The discovery of RALB followed shortly thereafter [16]. After the sequences of mammalian RALA and RALB were described, numerous RAL effectors were identified, including Ral-binding protein 1 (RALBP1) [17], Phospholipase-D1 (PLD1) [18], Filamin A [19], SEC5 [20], and EXO84 [21]. The two highly similar RAL paralogs play critical roles in many different cancer types [22,23]. However, despite decades of study, the functions of RALA and RALB in normal cell and cancer biology remain incompletely understood. In this review we highlight the overlapping and divergent roles of RALA and RALB in cancer, describe their effectors, examine their potential as therapeutic targets, and provide important considerations for future RAL-GTPase research.

## 2. Structure and Post-Translational Modifications of RAL-GTPases

Despite their overall similarities, important structural differences exist between the RALs. Here we review the structure, regulatory mechanisms and post-translational modifications of RALA and RALB to gain insight into the structural basis for their conserved and contradictory functions in cancer.

### 2.1. Structure

**Figure 1:**
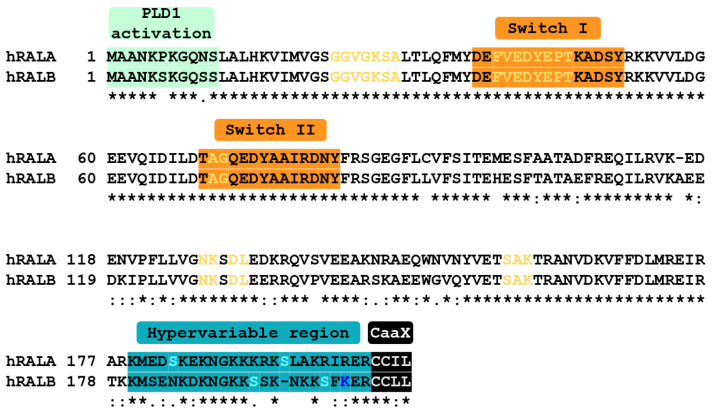
Comparison of human RALA and RALB sequences. Sequences were aligned using Clustal Omega. Residues fully conserved between RALA and RALB are marked by “*”, strongly conserved residues are denoted by “:”, while weakly conserved residues are marked by “.” N-terminal residues essential for PLD1 activation are highlighted in light green. Yellow lettering denotes those residues which define the guanine nucleotide binding pockets. Switch I and Switch II regions are highlighted in orange. Hypervariable regions are highlighted in turquoise. Light blue lettering within the hypervariable region denotes serine residues, which are known to be phosphorylated. RALB K200 (blue lettering) can be fatty acylated. Black highlighting denotes CaaX boxes which are sites of geranylgeranylation.

RALA and RALB share 82% sequence homology with one another and more than 50% sequence homology with RAS [16]. The first 11 amino acids of RALA and RALB are necessary for interaction with ADP-ribosylation factor 1 (ARF1) [24]. This interaction appears to be indirect [24]. As discussed in greater detail in Chapter 3, this N-terminal region is required for RAL-associated activation of phospholipase D [24,25]. Interestingly, the N-terminal region of RALA has only one serine residue at position 11 while the equivalent region in RALB has additional serines at positions 6 and 10 (Figure 1). It is not yet known if phosphorylation events at these residues are required for the formation of a complex with ARF1 and/or the subsequent activation of phospholipase D.

Residues 23–29, 39–46, 70–71, 127–128, 130–131, and 157–159 define the GDP/GTP binding pocket [26]. This pocket is conserved between RALA and RALB (Figure 1). Experimental mutation of the RALs at several key residues in the guanine nucleotide binding pocket has allowed for the generation of constitutively active and dominant-negative mutants. Q72L or G23V mutation locks RAL-GTPases in their GTP-bound, activated conformations [27,28]. Conversely, RAL G26A or S28N mutants are confined to their inactive, GDP-bound states [28,29].

Both RALs contain two switch regions which are a distinctive feature of small GTPases [30]. Switch I is located at residues 41–51 while Switch II is located at residues 69–81. These regions are fully conserved between RALA and RALB (Figure 1). Key downstream effectors of RALA and RALB interact with these Switch regions. These effectors include SEC5 and EXO84, members of the octameric exocyst complex [31], and RALBP1 [32,33]. Glutamate 38, alanine 48, and aspartate 49 are key residues for SEC5, EXO84, and RALBP1 binding, respectively [34,35]. These sites have been experimentally mutated to specifically uncouple the RALs from their effectors. The resulting mutant RALs have been proven to be invaluable tools for unraveling the precise contributions of each effector [23,34].

Most sequence differences between RALA and RALB are found in their C-terminal hypervariable tail regions, residues 179–206 for RALA and 180–206 for RALB (Figure 1). Within the hypervariable regions, positioning of serine residues as well as the number and positioning of lysine residues differ between the RAL GTPases. RALA has serine residues at positions 183 and 194, while in RALB serine residues are found at positions 192 and 198. Additionally, RALA has seven lysine residues while RALB has eight. The 8th lysine residue within the hypervariable tail of RALB can be fatty acylated, a modification not shared with RALA [36]. Resolved 3D structures of RALA [37,38] and RALB [39] suggest that GDP-RALA’s hypervariable tail is folded outward while the GDP-RALB’s hypervariable tail is folded inward [40]. Orientation of these regions is reversed in GTP-RALA and GTP-RALB [40]. The ramifications of these structural differences remain unclear but may influence protein function and localization.

### 2.2. Regulation of RAL Activation

Like other small G proteins, RAL activity is regulated by guanine nucleotide binding. Residues within the Switch I and Switch II regions interact with the gamma-phosphate group on GTP to lock small GTPases in their active confirmations. Hydrolysis of GTP to GDP allows the GTPase to relax into its inactive confirmation [30]. Members of the RALGEF and RAL GTPase-activating protein (RALGAP) families regulate RAL activity.

Interaction with RALGEFs occurs at the Switch I domain of the GTPase [5]. RALGEF binding promotes the dissociation of the bound guanine nucleotide and its replacement from the intracellular GDP/GTP pool. Because the intracellular GTP concentration is 10-fold higher than GDP concentration, this exchange favors RAL activation [40]. Known RALGEFs include RALGDS [41], RGL1 [42], RGL2 [43], RGL3 [44], RCC2 [28], RALGPS1 [45], and RALGPS2 [40,42,43]. RALGDS and RGLs 1, 2, and 3 share C-terminal RAS association (RA) domains and are RAS effectors [45]. RALGPS1 and RALGPS2 lack RA domains and are not RAS effectors [46]. RALGPS1 and RALGPS2 contain pleckstrin homology domains but their means of regulation are not well understood [47]. In cancer, elevated expression of numerous RALGEFs is associated with poor survival and increased local invasion and distant metastasis [48,49,50,51,52]. Downregulation of several RALGEFs has been shown to decrease the invasion and migration of cancer cells both in vitro and in vivo through decreased RALA or RALB activity [49,52,53,54,55,56,57,58]. Interestingly, RCC2 is the only RALGEF to interact with only RALA and not RALB, whereas there has been no evidence of preferential binding in the other RALGEFs [28].

The intrinsic ability of RAL GTPases to hydrolyze bound GTP into GDP is low [5]. RALGAPs are necessary to catalyze this reaction and return RAL activity to its basal state [59]. RALGAPs are heterodimeric complexes comprised of a shared RALGAPβ subunit and either RALGAPα1 or RALGAPα2 [60,61]. RALGAPβ complexes with RALGAPα1 and is designated as RALGAP1 while RALGAPβ with RALGAPα2 is designated as RALGAP2. These GAPs are thought to interact with the RALs through both the switch I and switch II domains [5,62,63]. Arginine residues in the GAP along with glutamine residues in the switch II region of the GTPase are necessary to catalyze GTP hydrolysis [5]. RALGAP activity is anti-tumorigenic. In several cancer subtypes, decreased expression of RALGAP1 and RALGAP2 correlates with worse overall survival and an increase in local invasion and distant metastasis [59,62,63,64]. Depletion or knockout of RALGAP1 or RALGAP2 increases invasion and migration of cancer cells, with a concomitant increase in RALA or RALB activation [59,62,63,64,65,66,67]. There is a lack of evidence for RAL paralog specificity among the RALGAPs.

Several pathways have been demonstrated to increase GTP-bound RALA, mainly through RAS activation. Ligands known to activate RALA through RAS include epidermal growth factor (EGF) [68,69,70], platelet-derived growth factor (PDGF) [68,71], endothelin [68], lysophosphatidic acid (LPA) [68], and insulin [68]. RAS-driven activation of RALB through these ligands has not been carefully investigated. Therefore, it remains unclear whether these extracellular signals can differentially activate RALA and RALB. RAS-independent activation of both RALA and RALB has been shown to occur in platelets through α-thrombin stimulating Ca^2+^/calmodulin binding to either RAL [68,72,73,74,75]. Clough et al. demonstrated that RALA and RALB both contain two calmodulin binding sites, an N-terminal Ca^2+^ independent binding domain and a C-terminal Ca^2+^ dependent binding domain [73]. In a yeast two-hybrid assay, RALA bound calmodulin more strongly than RALB [73]. Furthermore, truncation of RALB’s C-terminal tail increased its binding to calmodulin, while truncation of RALA had no impact on binding [73]. Work from Anirban Maitra and Barry Nelkin has demonstrated a potential role for cyclin-dependent kinase 5 (CDK5) as an important activator of both RALA and RALB, although the molecular mechanism of this regulation remains unclear [76].

### 2.3. RAL-GTPase Phosphorylation

RALA has two Aurora kinase A (AURKA) phosphorylation sites, serine residues S183 and S194, not shared with RALB [77]. These residues can be subsequently dephosphorylated by protein phosphatase 2A beta (PP2Aβ) [78]. Phosphorylation of RALA at S194 by AURKA has been shown to alter localization of RALA from the plasma membrane to the mitochondria and promote mitochondrial fission [79]. Given that S194 is not conserved between the RALs, this mechanism of mitochondrial translocation is not shared with RALB.

RALB also undergoes unique phosphorylation sites not shared with RALA. RALB has two serine residues located at S192 and S198, which are targets for protein kinase C alpha (PKCa) [80]. The identity of the phosphatase(s) that dephosphorylates these residues on RALB is currently unknown. Phosphorylation of RALB at S198 is required for migration in UMUC3 bladder cancer cells [23]. Additionally, phosphorylation of S198 was demonstrated to alter the localization of RALB. Upon stimulation with phorbol myristate acetate (PMA), RALB localization shifted from the plasma membrane to the perinuclear region. This event requires the ability of RALB S198 to be phosphorylated, as the RALB S198A mutant was unable to relocalize following PMA stimulation [23].

### 2.4. RAL-GTPase Ubiquitination

Protein ubiquitination can mark proteins for degradation, alter their localization, change their activity, and modulate their interactions [81]. There is evidence that ubiquitination of RAL-GTPases can alter their localization and function. RALA has 21 lysine residues for potential ubiquitination, but there is no consensus as to which of these lysine residues are most frequently modified [82]. RALB has six lysine residues that can potentially be ubiquitinated. Evidence suggests ubiquitination of K47 is a crucial determinant of RALB interaction with EXO84 or SEC5 [83]. Interestingly, when RALA and RALB are constitutively ubiquitinated, they demonstrate divergent subcellular localization. Ubiquitinated RALA localizes to the plasma membrane while ubiquitinated RALB concentrates in the cytoplasm, forming pronounced puncta [82]. The effects of ubiquitination on active GTP-bound states remains to be elucidated [82,83].

### 2.5. RAL-GTPase Geranylgeranylation

Many small GTPases are prenylated through the addition of farnesyl or geranylgeranyl groups. These modifications are critical for their localization and functions [84,85]. Both RALA and RALB C-terminal domains have CaaX boxes which are sites for geranylgeranylation [84]. Inhibition of geranylgeranyl transferases or mutation of the CaaX box disrupts RALA and RALB subcellular localization [84].

### 2.6. Post-Translational Modification Summary

RALA and RALB have several divergent post-translational modifications which may significantly alter their respective subcellular localization and function. However, much work remains to characterize the full spectra of modifications RALA and RALB may undergo and how these alterations regulate protein distribution and activity. Additionally, it is unclear how these post-translational modifications alter the binding affinities of critical effectors and regulatory components. One significant obstacle in addressing this knowledge gap is the lack of readily available tools for studying RALA and RALB phosphorylation. To date, there is only one commercially available phospho-RALA antibody, which recognizes phosphorylation at S194 (Millipore Sigma, St. Louis, MO, USA). There are no commercially available phospho-RALB antibodies, although one has been used and described in the literature [23].

## 3. RAL-GTPase Effectors

**Figure 2:**
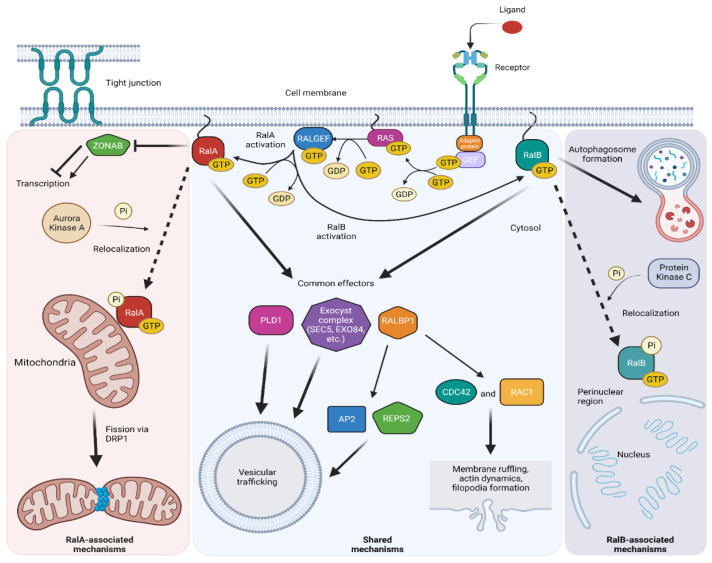
Mechanisms of RAL signaling. Active, GTP-bound RALA and RALB participate in a number of signaling pathways, which may be paralog-specific or common to both RALs.

As mentioned previously, the most comprehensively studied downstream effectors of the RALs are RALBP1, EXO84, and SEC5. In addition to these, several other downstream effectors are utilized by the RALs to influence cell behavior. Here, we will discuss in detail each of the known RAL effectors and highlight their roles in cancer (summarized in Figure 2).

### 3.1. RALBP1

The most studied RAL effector is RALBP1/RLIP76, which was discovered through two independent yeast two-hybrid screens [17,86]. RALBP1 contains two ATP-binding motifs and binds to the RAL switch regions [87,88]. RALBP1 is critical for RAL-mediated endocytosis, which will be discussed in detail in Section 4. In addition to its role in endocytosis, RALBP1 also regulates CDC42 and RAC1 activity. Here, RALBP1 functions as a GTPase-activating protein to these proteins [86,89]. CDC42 and RAC1 are small GTPases which regulate actin structure, filopodia formation and membrane ruffling [90]. Through specific interaction with RALA, RALBP1 has also been shown to be vital for mitochondrial fission during cell division. Kashatus et al. found that AURKA phosphorylates RALA to facilitate its relocalization to the mitochondria membrane [79]. In the mitochondria, RALA concentrates RALBP1 and dynamin-related protein 1 (DRP1) [79]. RALBP1 then recruits cyclin-B1/CDK1, promoting phosphorylation of DRP1 to facilitate mitochondrial fission [79]. Kashatus et al. also demonstrated that disruption of either RALA or RALBP1 leads to a failure in mitochondrial fission during mitosis [79].

Studies of RAL binding to RALBP1 and its downstream effects have largely focused on RALA in normal cellular physiology. Fenwick et al. showed that RALB can bind and complex with RALBP1 in a manner competitive with RALA [88]. In cancer cells, the RALB-RALBP1 interaction has been shown to promote invadopodia formation in pancreatic ductal carcinoma, with loss of RALB or RALBP1, disrupting the formation of invadopodia [91].

### 3.2. EXO84 and SEC5

Both RALA and RALB interact with two members of the exocyst complex, SEC5 and EXO84 [21,92,93]. This interaction is required for assembly of the exocyst, an octameric protein complex essential for polarized exocytosis [94]. SEC5 binds to the RALs at the switch I region, while EXO84 binds at both the switch I and the switch II regions [20,21]. The RAL paralogs do not appear to preferentially bind either exocyst subunit, and in one study depletion of both paralogs was required to decrease exocytosis [95]. Interestingly, RALA and RALB bind to SEC5 and EXO84 in a competitive manner and RNAi-mediated knockdown of RALA leads to the exocyst localizing to the perinuclear compartment of cells, while knockdown of RALB leads to the exocyst accumulating in large cytoplasmic vesicles [34,96]. The RALs influence invasion and migration of cancer cells through exocyst-driven filopodia formation and release of MMPs to degrade the extracellular matrix [97]. RALB has been implicated as the more important RAL paralog for filopodial invasion and migration in normal rat kidney cells [98], B cells [99], multiple myeloma [99], pancreatic UMUC-3 cells [97] and prostate DU145 cells [97]. In these cells, RALA depletion resulted in no change in migration, while RALB depletion consistently resulted in reduced migration. Expression of constitutively active RALA G23V in UMUC-3 bladder cancer cells significantly reduced cell motility whereas RALB G23V stimulated motility significantly [97]. These studies clearly demonstrate a role for both RALs in vesicular trafficking through the exocyst and suggest that RALB may be the more important paralog in the context of cancer invasion and migration.

### 3.3. PLD1

PLD1 catalyzes the hydrolysis of phosphatidylcholine to phosphatidic acid in response to extracellular activation of tyrosine receptors [100]. Jiang et al. first showed that RALA associates with PLD1 in vitro by precipitating RALA complexed with PLD1 in normal and Src-transformed cells [25]. Interestingly, point mutations which uncouple RALA from RALBP1, or inactive GDP-RALA, still bind PLD1 [24]. RALA acts synergistically with another small GTPase—ARF1—to activate PLD1 [18,24]. Both RALA and ARF1 interact with PLD1 but these interactions occur at unique sites on PLD1 [18,24]. Association of ARF1 and RALA, as well as RALA-associated activation of PLD1, requires RALA’s N-terminal 11 amino acids [18,24]. A complex of RALA, ARF1, and PLD1 is important for vesicular trafficking between the plasma membrane, Golgi apparatus, and endoplasmic reticulum [101]. While many studies of PLD1 activation by the RALs focus on RALA, PLD1 is also an effector of RALB. Rybko et al. found that disruption of the RALB-PLD1 interaction decreases the metastasis of HET-SR cells in a hamster tumor model, whereas decoupling mutants for RALBP1 and SEC5/EXO84 did not decrease metastasis [102]. More recently, Ghoroghi et al. discovered that RALA or RALB depletion in 4T1 cells causes a significant decrease in PLD1 localization on the plasma membrane and reduces exosome secretion to the same extent as PLD1 inhibition [103]. These studies show that PLD1 is a direct effector of both RALA and RALB involved in vesicular trafficking and exosome secretion.

### 3.4. Filamin

Filamin is an actin filament-crosslinking protein which interacts with CDC42 to induce filopodia formation in fibroblasts. Ohta et al. showed that RALA interacts with filamin in a GTP-dependent manner to induce filopodia formation in 3T3 fibroblast cells [19]. This interaction is absent in filamin-deficient melanoma cells and can be rescued by the addition of filamin 1 in these cells [19]. While these activities have been demonstrated for RALA, RALB has not yet been shown to interact with filamin or alter actin through this effector pathway. Investigating these interactions may shed light on how the RAL paralogs alter actin organization and influence cancer cell migration.

### 3.5. Autophagosome

The autophagosome, the key vesicular structure of macroautophagy, fuses with lysosomes during the autophagic degradation and recycling of cellular contents [104]. Bodemann et al. showed that depletion of RALB and not RALA inhibited autophagosome formation in HEK293A GFP-LC3 cells [105]. Furthermore, this group found that RALB was recruited to the site of autophagosome assembly and drives the assembly of the autophagosome by recruiting and interacting with EXO84 and Beclin1 [105]. Ubiquitylation of RALB caused a reduction in interactions with EXO84 and increased SEC5 binding, reducing autophagosome formation, while deubiquitination of RALB restored RALB-EXO84 binding and autophagosome formation [83]. RALA was also investigated in each of these studies and was found to be dispensable to autophagosome function.

### 3.6. JNK/Jun Pathway

The C-Jun N-terminal Kinase (JNK) pathway regulates cell proliferation, growth, differentiation, survival, and apoptosis. The RAL paralogs are regulators of the JNK signaling pathway. In *D. melanogaster*, Sawamoto et al. found that constitutively active Rala causes improper dorsal closure in vivo, a process known to be affected by alterations in JNK signaling [106]. In addition, they found that constitutively active Rala decreased JNK phosphorylation in S2 cells in vitro, with Rala acting as a negative regulator of the JNK pathway [106]. This result was confirmed by Balakireva et al. and was shown to be conserved in mammals through siRNA depletion of RALA in HeLa cells, leading to an increase in C-Jun and JNK phosphorylation after induction with insulin [107]. Here, RALB was not tested because its depletion caused cell death. In studies of RAS-mediated phosphorylation of c-Jun, dominant negative forms of RALA or RALB abolished RAS-dependent phosphorylation of C-Jun in A14 and HEK-293 cells [108]. Collectively, these studies demonstrate an evolutionarily conserved role for the RALs in regulation of the JNK pathway, which is redundant between the mammalian paralogs.

### 3.7. ZONAB

The ZO-1-associated nucleic acid-binding protein (ZONAB) is a Y-box transcription factor that regulates proliferation and is involved in the intracellular response to cell density [109]. ZONAB can function as a transcriptional repressor [109] and activator [110], and its activity is itself regulated through binding interactions with ZO-1, a protein associated with tight junctions that sequesters ZONAB outside of the nucleus [109]. Active RALA interacts with ZONAB’s cold shock domain, which is also used for DNA binding [111]. Interestingly, the degree of RALA:ZONAB interaction increases with cell density, which coincides with the relocalization of RALA from the cytoplasm to the cell membrane at the high density [111]. Transactivation assays in Madin-Darby canine kidney (MDCK) cells revealed that constitutively active Q72L RALA halted ZONAB-mediated transcriptional repression, while blocking RALA increased the repressive activity of ZONAB [111]. Thus, increased RALA activation can block ZONAB-driven transcriptional regulation by sequestering it at tight junctions [111]. It is not known whether ZONAB also interacts with RALB or how this interaction may influence cell behavior.

## 4. Divergent Roles of RALA and RALB in Endocytosis, Exocytosis, and Vesicle Trafficking

As mentioned previously, interaction between the RALs and RALBP1 facilitates endocytosis, while the RALs cooperate with SEC5 and EXO84 to mediate exocytosis. Altered endocytosis and exocytosis are hallmarks of cancer progression [112,113]. However, precisely how RAL-mediated vesicular trafficking contributes to cancer aggressiveness and metastasis remains poorly understood. Furthermore, how competition between the RALs for RALBP1, SEC5, and EXO84 binding influences trafficking remains an important unanswered question. We next delve more deeply into the contributions of the RALs during vesicular trafficking.

### 4.1. Endocytosis

The RALs, through their effector RALBP1, are important regulators of endocytosis, a process frequently disrupted in cancer [114]. Endocytic trafficking is vital for the homeostasis of a number of tyrosine kinase receptors, including EGFR [115] and PDGFR [116]. Malignant cells tend to favor recycling of receptors, rather than lysosomal degradation, in order to perpetuate signaling [114]. Additionally, internalization of adherence junctions and tight junction proteins promotes the loss of cell–cell contacts and promotes cell migration [114].

The interaction between RALA and RALBP1 was discovered by Feig et al. in 1996 [17] but it was not until later that RALBP1 was shown to facilitate receptor-mediated endocytosis of EGF [117]. Nakashima et al. demonstrated that the C-terminal region of RALBP1 interacts with REPS2 (RALBP1 Associated EPS Domain Containing 2), a known regulator of receptor-mediated endocytosis with EPS homology domains, which regulate the binding and internalization of EGFR through Epsin and EPS15 [117]. The N-terminal domain of RALBP1 also interacts with adaptor protein complex 2 (AP2) to mediate clathrin-dependent endocytosis of receptors [35]. RALBP1 dissociates AP2 from the AP1 complex at the Golgi and then transports AP2 to the plasma membrane to perform receptor-mediated endocytosis. Beyond interacting with RALBP1 to regulate endocytosis, it has been shown that RALA is required for caveolae-mediated endocytosis by stimulating PLD2 generation of phosphatidic acid [118]. EGFR was demonstrated to require RALA for internalization during intestinal stem cell maintenance in *D. melanogaster* [119]. In 2009, Han et al. demonstrated RALA activation and translocation of RALBP1 promotes NMDA receptor-dependent AMPA receptor endocytosis in postsynaptic neurons [120]. Activation of the NMDA receptor triggers a signaling cascade, leading to activation of RALA, which in turn interacts with RALBP1 to translocate to the membrane where RALBP1 is dephosphorylated by the NMDA receptor. RALBP1 may then bind to PSD-95, allowing endocytosis of the NMDA receptor [120].

Because initial studies of RALBP1 focused solely on interactions with RALA, individual contributions of the RAL paralogs during endocytosis remains to be fully elucidated. In the first study to explore a role for RALB interaction with RALBP1 in endocytosis, Jullien-Flores et al. showed expression of constitutively active RALB G23V decreased the internalization of EGFR and the transferrin receptor [35]. In one of the few studies to consider the contributions of both RAL paralogs during receptor endocytosis, Johansson et al. found that both RALs mediate the internalization of Frizzled-7 in mouse intestines [121]. Loss of RALA or RALB decreased Frizzled-7 internalization and disrupted Wnt signaling, leading to improper intestinal crypt formation, and complete loss of both RALs led to death of the intestinal crypts [121].

### 4.2. Exocytosis

In addition to regulating endocytosis, the RALs also regulate vesicular trafficking through exocytosis. Through this process, the RALs influence polarization of epithelial cells, which is important for the proper spatial expression of membrane proteins and secreted growth factors [122]. Improper localization of cell surface receptors like EGFR to the apical or basolateral membrane results in inappropriate exposure to growth factors [123]. Exocytosis of MMPs is also crucial for cancer invasion and metastasis. Likewise, formation of invadopodia and filopodia depend on exocyst function [69,97,98,99,124,125]. The exocyst is also important for proper cell division and its dysfunction can lead to genomic instability, a hallmark of cancer [126].

In pancreatic islet β cells, RALA is necessary for the release of insulin granules in response to low glucose [127]. When constitutively active G23V RALA is expressed, insulin secretion by pancreatic islet β cells is increased, while dominant negative S28N RALA reduces insulin secretion [127]. RALA not only aids in insulin release, but Chen et al. demonstrated that RALA associates with GLUT-4-containing vesicles in adipocytes [128]. RALA was found to be activated by insulin in both a dose- and time-dependent manner, and knockdown of RALA in these cells significantly reduced the uptake of glucose upon insulin stimulation. Following insulin stimulation, RALA is bound to GTP in adipocytes and interacts with the exocyst complex and MYO1C to traffic GLUT-4-containing vesicles to the plasma membrane and increase glucose uptake [128]. Here, a clear role for RALA in insulin release and trafficking of GLUT4 receptors is apparent, but RALB has not yet been evaluated in these processes. Additionally, RALA was found to be important for controlling glucose homeostasis [129]. While GLUT4 is associated with metastasis of head and neck squamous cell carcinoma [130] and is involved in EMT [131], a specific role for RALA-mediated GLUT4 trafficking in cancer has not yet been established. It also remains unclear what roles, if any, RALB may play in these mechanisms.

In epithelial cells, RALA, but not RALB, is integral to the delivery of vesicles to the basolateral membrane [31,132]. Early work by Moskalenko et al. demonstrated that expression of RalB23V, a constitutively active form of RALB, results in improper trafficking of EGFR to both the basolateral and apical membranes and disrupts the localization of vesicular stomatitis virus G protein in MDCK cells [21]. Shipitsin and Feig expressed constitutively active Q72L RALA or Q72L RALB in MDCK cells and found that Q72L RALA increased exocyst-driven delivery of E-cadherin and EGFR to the basolateral membrane [132]. On the other hand, Q72L RALB did not increase exocytosis of E-cadherin or EGFR to the plasma membrane. Immunofluorescence of these constitutively active RALs revealed distinct subcellular locations of RALA and RALB in MDCK cells, with RALA highly co-staining with SEC5 at the basolateral membrane [132]. RALB did not highly co-localize with SEC5 and was significantly reduced at the plasma membrane compared to RALA, instead being found diffusely throughout the cytoplasm of the cell [132]. Loss of cellular polarity can lead to abnormal delivery of cellular proteins or ligands to the incorrect membrane, leading to cancer initiation or progression. In MDCK cells, EGFR is normally expressed on the basolateral membrane, but small pools of EGFR are also found on the apical side [133]. Singh et al. showed that epiregulin can bind and transiently activate basolateral EGFRs, but when epiregulin is improperly delivered to the apical membrane through deletion of the carboxy-terminal 25 residues, there is prolonged activation of EGFR, leading to uncontrolled proliferation [133]. Thus, loss of polarity due to improper trafficking of cell surface proteins can lead to aberrant signaling in cancer. These studies demonstrate a critical role for RALA during the delivery of vesicles to the basolateral membrane which is not shared with RALB.

During cytokinesis, RALA localizes to recycling endosomes through its interaction with the exocyst [134]. In *D. melanogaster*, RALA is necessary for cleavage furrow formation during cytokinesis [135]. Holly et al. developed RALA-deficient flies using a *Rala^PL56^* germline clone and found that when RALA is deficient there is no formation of the cleavage furrow and a failure of cytokinesis. In mammalian Cos-1 cells, Chen et al. demonstrated that RALA can localize to the pericentrosomal membrane and at the centrosome during mitosis using a fluorescently labeled peptide of the RAL binding domain to SEC5 [134]. This group further demonstrated that RALA localized with the transferrin receptor and RAB11 in recycling endosomes and not with the Golgi or early endosomes [134]. Crucially, disruption of the RALA-SEC5 interaction caused cytokinesis to fail, resulting in binucleation of Cos-1 and HeLa cells [134]. RALB also has a role in recycling endosomes and cytokinesis, but it is distinct from that of RALA. Cascone et al. found that RALA localizes to the cleavage furrow during cytokinesis, in agreement with Holly et al. and Chen et al. However, when evaluating RALB, this group found that RALB is absent in early cytokinesis but is found at the midbody during the late stages of cytokinesis [136]. Given that failure of cytokinesis during division results in chromosomal instability and aneuploidy [137,138,139,140], dysregulated cytokinesis represents yet another mechanism through which the RALs may contribute to cancer initiation and progression.

### 4.3. Exosome Secretion

Exosomes have emerged as critical mediators of tumor progression [141,142,143]. Exosomes secreted by primary tumor cells are important contributors to formation of the pre-metastatic niche [144,145,146]. Additionally, secreted extracellular vesicles have been demonstrated to alter the metabolism [147] and signaling [148,149] of cells in the tumor microenvironment (TME) to promote cancer growth and progression. Both RAL GTPases have been implicated in the biogenesis and secretion of exosomes in normal cell biology [150,151] as well as in cancer [103]. Interestingly, RAL GTPases control exosome secretion through interactions with PLD1 [152] rather than the exocyst complex. Ghoroghi et al. demonstrated that RALA and RALB can be found within exosomes in the breast cancer cell line 4T1 [103]. Whether exosome-transported RAL proteins contribute to the creation of a supportive TME or help cultivate the premetastatic niche remains to be elucidated.

## 5. Divergent Roles of RALA and RALB in Cancer

The RAL-GTPases can have redundant, compensatory, or divergent roles in a cancer-dependent or even cell line-dependent manner. The molecular basis for this variation largely remains an enigma. We will next review RAL-GTPase functions in a number of cancer types.

### 5.1. Bladder Cancer

Substantial literature on RAL-GTPases in bladder cancer has been published, supporting divergent roles for RALA and RALB. Early work from Oxford et al. showed elevated GTP-RALA levels in the bladder cancer cell lines UMUC-3 and DU145 [97]. When siRNAs specific to RALA or RALB were used in transwell assays, only RALB was found to be required for migration [97]. Constitutively active RALA inhibited migration, while constitutively active RALB promoted transwell migration. Smith et al. later demonstrated that both RALA and RALB are greatly upregulated in invasive bladder cancer relative to normal cells [153]. Additionally, the RAL effector RALBP1 was significantly upregulated in invasive cancer samples. The importance of RALB in bladder cancer was later reinforced by important work from Dan Theodorescu’s group, which demonstrated that phosphorylation of RALB by protein kinase C (PKC) was required for bladder cancer tumor growth and experimental lung metastasis of the metastatic bladder cancer cell line UMUC-3 [23].

### 5.2. Blood Cancers

Early evidence demonstrated that chronic myeloid leukemia (CML) cells have increased RALA expression, and depletion of RALA with siRNAs reduced cell proliferation and increased caspase 3 expression [154]. Additionally, siRNA targeting of RALA sensitized CML cells to arsenic compounds [154]. Another study observed elevated RALA activity in CML cell lines and patient samples [155]. This study also found overexpression of RALA in K562 and BaF3 cells induced progression and resistance to imatinib [155]. Importantly, inhibition of RALA by RBC8, siRNA, or miR-181 attenuated CML malignant phenotypes and sensitized cells to imatinib [155]. However, neither of these studies addressed a potential role for RALB in CML.

In acute myeloid leukemia (AML), depletion of RALB was shown to phenocopy loss of NRAS(V12), while loss of AKT or MAPK did not [156]. Loss of RALB was also found to decrease TBK1 phosphorylation and reduce BCL2 expression [156]. Other work in AML suggests that RALB plays a critical role in relapse of NRAS(V12)-independent (NRI) AMLs [157]. Importantly, indirect inhibition of RALB via the CDK5 inhibitor dinaciclib induced apoptosis in NRI-AML cell lines in vitro and reduced the tumor burden in AML PDX models in vivo [157]. These AML studies, like the previously described CML studies, focused on only one RAL paralog.

Early work in multiple myeloma (MM) suggests that RALB is important for migration in the OPM-1 and NCI-H929 cell lines [99]. Additionally, treatment of MM cells with stromal-derived factor 1 induced migration, where RALB rather than RALA was found to mediate this migration [99]. In contrast, more recent work has suggested that both RALs may contribute to MM [158]. In these experiments, both RAL paralogs were found to be strongly expressed in MM cell lines [158]. Loss of either RALA or RALB decreased MM cell line viability, although depletion of RALA was associated with greater cell death and RALA alone was required to sustain AKT activity [158]. Viability of MM cell lines after treatment with the dual RAL inhibitor RBC8 was variable, with INA-6 cells showing sensitivity to the drug while MM.1S cells were resistant [158].

### 5.3. Breast Cancer

The earliest studies exploring RAL-GTPases in breast cancer suggested that RALA may be involved in tamoxifen resistance in breast cancer cells [159] and EGFR-independent growth [160]. Recently, several important studies have been published describing the roles of the RALs in breast cancer with contradictory results. Our lab recently demonstrated that RALA and RALB appear to have divergent roles in the triple negative breast cancer (TNBC) cell line MDA-MB-231 [22]. RALA, but not RALB, was required for MDA-MB-231 tumor growth, invasion, and metastasis. Additionally, we found RALA expression, but not RALB expression, to be prognostic of patient outcome and predictive of patient response to chemotherapy [22]. Importantly, inhibition of both RALA and RALB with the nonselective RAL inhibitor BQU57 significantly reduced tumor growth and metastasis in the TNBC cell line MDA-MB-231 and patient-derived xenograft models [22]. Recent work by Vincent Hyenne’s group has found a critical role for RALA and RALB in extracellular vesicle trafficking, driving breast cancer metastasis [103,150]. In these studies, depletion of RALA or RALB in the murine 4T1 cell line reduced exosome production and secretion. Interestingly, depletion of RALA increased the growth of 4T1 primary tumors, while depletion of RALB slowed primary tumor growth. However, knockdown of either RALA or RALB reduced spontaneous lung metastasis of 4T1 cells [103]. Another recent study found a correlation between RALB protein expression and progression toward metastasis in human breast cancer samples [53].

### 5.4. Colorectal Cancer

Important work from Channing Der and colleagues demonstrated differing roles for the RAL GTPases in colorectal cancer. It was demonstrated that colorectal cancer cell lines have significantly increased GTP-RALA and total RALA expression relative to normal adjacent mucosa [161]. GTP-RALB and total RALB expression in these samples were only somewhat increased relative to normal adjacent mucosa [161]. Additionally, knockdown of RALA by shRNA greatly decreased anchorage-independent growth regardless of KRAS or BRAF mutation status, while RALB knockdown increased growth [161]. This work highlights the sometimes paradoxical roles of RALA and RALB in cancer. Recent experiments by Khawaja et al. demonstrated that knockdown of RALB by siRNA decreased cell viability, while knockdown of RALA moderately increased cell viability regardless of KRAS mutation status [162]. Elevated RALB mRNA expression was also associated with poor prognosis [162].

### 5.5. Gastric Cancer

Studies by Wang and colleagues demonstrated that RALA interacts with RCC2 in SGC-7901 and MGC-803 human gastric cancer cells [163]. Loss of RCC2 decreased RALA activity, and inhibition of RALA via the non-specific RAL inhibitor RBC8 reduced proliferation, migration, and invasion of gastric cancer cell lines [163]. A role for RALB was not investigated in this study [163]. In another study, approximately 15% of gastric cancer patients were found to have RALA autoantibodies [164]. The presence of these antibodies did not correlate with either stage or survival [164].

### 5.6. Hepatic Cancer

The importance of RAL GTPases in hepatic cancer was first described by Wang and colleagues [165]. Here, RALA expression and RALA autoantibody levels were found to be significantly higher in tissue and sera samples from HCC patients relative to patients with liver cirrhosis or normal controls [165]. These initial observations of an important role for RALA in HCC were further supported by work from Ezzeldin et al. which demonstrated increased GTP-RALA in HCC tissue samples and cell lines [166]. RALA silencing greatly decreased cell proliferation and invasion in vitro [166]. In addition, HCC cell populations enriched for the cancer stem cell marker CD133 were found to have significantly increased expression of both total RALA and active GTP-RALA [166]. These early investigations of RAL GTPases in hepatic cancer focus exclusively on RALA, without investigating the possible contributions of RALB. More recent investigation of both RAL GTPases also supports the unique importance of RALA in HCC. RALA, but not RALB, was found to be significantly upregulated in HCC relative to non-tumor tissues [62]. The expression of RALA increases with cancer stage and high RALA is associated with worse overall survival [62]. Additionally, knockdown of RALA decreased stemness and migration while overexpression of RALA amplified these features [62].

### 5.7. Lung Cancer

Experiments performed by Male and colleagues focused on the role of RALA in lung cancer [167]. Here, siRNA inhibition of RALA significantly increased apoptosis and necrosis of lung cancer cell line A549 and reduced invasion [167]. In addition, sorting non-small cell lung cancer (NSCLC) cells using the cancer stem cell marker CD44 revealed elevated GTP-RALA levels in the CD44^high^ putative stem cell population compared to CD44^low^ populations [167]. In this study, RALB was not investigated. Christopher Marshall’s group used conditional knockout of the RAL alleles in a *KRAS*-driven mouse model of lung carcinoma [168]. These experiments revealed redundant roles for RALA and RALB. Either paralog was sufficient to sustain tumor growth and only when both RALA and RALB were deleted was tumor formation blocked. Experiments performed by Santos et al. suggest RALB has a novel cell cycle regulatory role not shared with RALA [56]. Using a large panel of NSCLC cell lines with and without KRAS mutations, transient knockdown of RALB disrupted cell cycle progression resulting in arrest in G0/G1. This phenotype was not shared following the knockdown of RALA. Biondini et al. showed that RALB mediates contractility and cancer dissemination upon TGFB stimulation [169]. This feature was not found to be shared with RALA, providing further evidence that cell migration in lung cancer appears to be a RALB driven phenotype [169]. Spiegelman et al. found that RALB lysine fatty acylation is required for proper cell migration, membrane localization, and association with exocyst complex components SEC5 and EXO84 [36]. However, it is unclear if this effect is truly specific to RALB, as RALA was not rigorously investigated.

### 5.8. Melanoma

Early investigations of the RAL GTPases in melanoma have suggested that RALA is the predominant paralog. Assessment of the levels of active GTP-bound RALA and GTP-bound RALB demonstrated that independently derived melanoma cell lines have much higher GTP-RALA levels and essentially absent GTP-RALB [170]. In a panel of independently derived melanoma cell lines, there did not appear to be an association between mutant NRAS, mutant BRAF, and/or elevated GTP-RAL. In vivo work suggests that depletion of either RAL-GTPase with shRNA significantly reduces tumor growth in systems with wild type or mutant NRAS or BRAF [170]. 

### 5.9. Nerve Sheath Tumors

Bodempudi et al. first described global activation of RALA in malignant peripheral nerve sheath tumor (MPNST) cell lines [171]. Their work also demonstrated that expression of an inhibitory RALA S28N mutant or silencing of RALA by siRNA decreased the invasiveness of MPNST cell lines. However, RALB was not investigated in this study. Work by Gandapathy and Fagman et al. investigated both RALA and RALB in MPNST and found divergent roles for the paralogs [172]. RALA, but not RALB, was found to be active and associated with RALBP1 in NF1-deficient MPNST cells [172]. The authors speculate that RALA is activated under NF1-deficient conditions and may be negatively regulated by PKC. Inhibition of PKC in NF1-deficient MPNST cells resulted in Chk1 nuclear translocation and eventual apoptosis in a RALA-dependent manner [172].

### 5.10. Ovarian Cancer

Both RALA and RALB are highly upregulated in ovarian cancer cell lines [173]. However, only levels of active GTP-bound RALA, and not GTP-RALB, are elevated in cancerous cells relative to normal cells [173]. Inhibition of RALA greatly reduced ovarian cancer cell growth and invasion in vitro and tumor growth in a mouse model [173]. More recently, Gong et al. demonstrated that RALA and RALBP1 are significantly upregulated in cisplatin-resistant ovarian cancer [174]. The same group described the involvement of RCC2, an upstream effector of RAL, and RALA in ovarian cancer cell proliferation, migration, and cisplatin resistance [174]. RCC2 promotes tumorigenesis through increased RALA signaling mediated by a direct protein–protein interaction, identified by co-immunoprecipitation [174].

### 5.11. Pancreatic Cancer

Both RALA and RALB are highly expressed in pancreatic adenocarcinoma [175]. Lim et al. demonstrated that RALA, and not RALB, was required for the establishment of pancreatic cancer cells in mouse models [176]. Later, it was found that RALA, but not RALB, supports anchorage-independent growth in the pancreatic cancer cell line MiaPaCa2 [84]. In experiments utilizing PANC-1 cells, RALB was shown to be an important part of a multiprotein complex which drives tumor stemness and treatment resistance [177]. Additional investigations support the idea that RALB plays a more important role in pancreatic cancer progression than RALA. Neel et al. showed that the interaction of RALB and RALBP1 was required for invadopodia formation in multiple pancreatic cancer cell lines [91]. Oeckinghaus et al. discovered NF-κB inhibitor interacting RAS-like proteins 1 and 2 (κB-Ras1 and κB-Ras2), which interact with the RALGAPs and are required for their GAP function [175,178]. This group has shown that κB-Ras is frequently decreased in pancreatic ductal adenocarcinoma (PDAC) patients and loss of κB-RAS cooperates with oncogenic RAS to drive cancer initiation and progression in a PDAC mouse model [175]. In other studies, the small noncoding RNA miRNA-139 was found to negatively regulate RALB through a RAL/RAC/PI3K pathway in order to prevent pancreatic cancer in mouse models [179].

### 5.12. Prostate Cancer

In prostate cancer, the relative importance of the RAL GTPases remains unclear. Neither RALA nor RALB were found to be required for primary tumor growth of PC3 cells but both paralogs contributed to bone metastasis [180]. It has also been demonstrated that Myc-associated zinc finger protein (MAZ) promotes prostate cancer metastasis to the bone through transcriptional upregulation of K-RAS and subsequent activation of RALGEF and RALA [181]. Several other investigations in prostate cancer have focused largely on RALA without complementary investigation of RALB. RALA was demonstrated to regulate VEGF-C expression in prostate cancer cells undergoing androgen ablation [182,183]. Hazekett and Yeaman demonstrated that the interactions between RALA and the exocyst complex are required for migration and invasion of PC-3 prostate cancer cells [93]. Later, Li et al. showed that anti-RALA autoantibodies were overexpressed in prostate cancer regardless of subtype, pathology, clinical stage, or Gleason Score [184]. Chen et al. showed that knockdown of RALA in PC-3 prostate cancer cells inhibits cell proliferation, enhances apoptosis, inhibits migration and invasion, and is required for the localization of AQP3 [185].

### 5.13. Renal Cancer

Little is known about the role of RAL GTPases in renal cancer. Li et al. demonstrated that RALA was required for invasiveness in human SN12C and HK2 cells using siRNA [186]. Here, knockdown of RALB did not mimic RALA depletion, suggesting unique contributions for the two paralogs in this setting [186]. Given the importance of the exocyst complex and epithelial tight junctions in renal function [187], it is likely that the RALs have important undiscovered roles in renal physiology, pathology and cancer.

### 5.14. Summary of RAL-GTPases in Cancer and Considerations for Future Research

The diverse functions for RALA and RALB reported across the cancer literature are summarized in Table 1. RALA and RALB can have redundant roles, such as in KRAS-driven lung carcinoma [53]. Conversely, RALA and RALB can have discrete functions as demonstrated in breast [22], bladder [97], and colorectal cancers [167,188]. To further complicate matters, these divergences are not consistent across cancer types or even across cell lines within a cancer type. For example, RALA is required for invasion of MDA-MB-231 breast cancer cells [22], but in bladder cancer RALB is found to support invasion [23,153]. Even more confoundingly, RALA supports tumor growth of MDA-MB-231 cells while suppressing primary tumor growth in the similar 4T1 line [22,103]. The precise molecular contexts which support the redundant or divergent roles of RALA and RALB remain to be elucidated. More comprehensive analyses of the interactions between the RAL-GTPases and their shared pool of effectors may shed light on this enigma. In addition, a better understanding of the full spectra of post-translational modifications of RALA and RALB and how these modifications alter subcellular localization and effector utilization is required to provide a framework for their various roles in cancer.

Inconsistencies in study design further limit our understanding of the distinct roles of the RALs in cancer. Many studies have focused on only one RAL paralog, neglecting to study the other, or even failing to indicate which paralog was investigated. Additionally, the similarity of the RALA and RALB sequences have made it difficult to study one specifically without potentially confounding effects from unintentionally targeting the other paralog. Unpublished work from our group has found several commercially available antibodies and interfering RNA sequences intended to explicitly detect or target RALA or RALB are in fact not specific. Careful and thorough validation of antibodies and targeting RNAs is imperative if we are to unravel the independent functions of RALA or RALB across cancer.

## 6. Therapeutic Targeting of RAL-GTPases

Given their importance in cancer biology, RALs have emerged as desirable clinical targets. Unlike many other members of the RAS superfamily, the RAL structure is amenable to drug targeting [40]. Early efforts to indirectly target the RALs have recently given way to the development of direct inhibitors. Below we will describe these endeavors and provide a commentary on the future of RAL-targeting therapies.

### 6.1. GGTase-I Inhibitors

The first attempts to inhibit the RALs focused on targeting the geranylgeranylation of both RALA and RALB paralogs. Falsetti et al. expressed farnesylated C-terminal domains of RALA or RALB in Cos7 cells and MiaPaCa2 pancreatic cancer cells to shield them from GGTase-I inhibitors (GGTIs) [84]. Treatment of MiaPaCa2 cells with farnesyl-RALA protected them from the inhibitory impact of GGT-I-2417 on anchorage-dependent growth. Farnesylated RALB protected GGT-I-2417-treated cells not only from inhibition of anchorage-dependent growth, but also from apoptosis measured using a TUNEL assay [84]. The impact of GGTIs upon the RAL function has been studied in other cancer types, including pancreatic cancer [189], oral squamous cell carcinoma [190], hepatocellular carcinoma [166] and medulloblastoma [191]. GGTIs remain an active area of research but are limited in clinical application due to their lack of specificity.

### 6.2. Indirect Inhibition

In addition to GGTIs, other attempts have been made to indirectly inhibit the RAL paralogs by targeting their upstream activators. Dinaciclib is an inhibitor of CDK5, an activator of the RALs, shown to reduce pancreatic cancer initiation, progression and metastasis in mouse xenograft models by reducing activation of RALA and RALB [76,192]. However, despite showing promise in preclinical models, dinaciclib failed to provide clinical benefit in phase I trials [193]. AURKA is a target for preferentially inhibiting RALA activation. As mentioned previously, AURKA activates RALA by phosphorylating RALA serine residues S183 and S194, which are not shared with RALB. Alisertib is an AURKA inhibitor that disrupts spindle formation in dividing cells and is effective in reducing tumor proliferation in mouse models [194,195,196]. Inchanalkar et al. delivered alisertib in polysaccharide nanovesicles and found that this treatment reduced anchorage-independent growth of MCF-7 breast cancer cells in 2D and 3D cell cultures, specifically through the reduction of activated RALA [197]. Despite early promise, alisertib failed Phase III clinical trials in 2015 and has not received FDA approval as of 2022.

### 6.3. Small Molecule RAL Inhibitors

In 2014, Yan et al. discovered small molecule inhibitors capable of binding an allosteric site accessible in the inactive GDP-bound forms of RALA and RALB. These compounds—RBC6, RBC8, RBC10, and BQU57—are thought to stabilize the RALs in their inactive confirmations [198] and have been tested preclinically in several cancer models. RBC8 has been shown to reduce growth of chronic myelogenous leukemia [155], gastric cancer cells [163], and hepatocellular carcinoma [62]. RBC8 and BQU57 have both been observed to inhibit lung cancer growth and bladder cancer in soft agar and in vivo [198]. Work by Oeckinghaus et al. has also demonstrated that BQU57 decreases anchorage-independent growth of PDAC cells in vitro [175]. More recently, our group has studied the effects of BQU57 and found it reduced both primary tumor growth and metastatic growth in vivo in a TNBC cell line and patient-derived xenograft models [22]. The ability of these small molecules to block growth of both primary and metastatic lesions highlights the enormous potential of RAL-targeting therapies. How non-selective RAL inhibitors may be best combined with current standard of care treatments is an important but outstanding question. Our recent data suggest that RALA may provide TNBCs with resistance to chemotherapy and that combination treatment with RAL inhibitors may overcome this resistance [22]. Given the frequently non-redundant roles of RALA and RALB in cancer, the development of paralog-specific inhibitors would provide the field with invaluable research tools and potentially powerful therapeutics.

## 7. Conclusions

Research on RAL GTPases in cancer continues to clarify their important roles in many cancer subtypes and reinforce their attractiveness as therapeutic targets. Most evidence supports RALA as the more important RAL GTPase in ovarian cancer and prostate cancer, while RALB appears to be more important for cancer progression and metastasis in bladder and lung cancers. In many other cancers, the relative importance of each RAL paralog remains elusive. For those contributing to RAL-related cancer research, it is critical that studies rigorously address the contribution of both paralogs and account for potential confounding effects caused by the substantial similarities between RALA and RALB. Only through continued efforts to fully unravel the RAL enigma will these small G proteins reach their potential as clinical targets.

## Figures and Tables

**Table 1:** Roles of RALA and RALB in specific cancer subtypes.

Cancer Subtype	RALA Role	RALB Role
Bladder cancer	RALA inhibits migration [97]	Phosphorylation of RALB is required for tumor growth and metastasis [23] and migration [97]
Blood Cancers	Upregulated and supports proliferation in CML [154]	May promote AML colony formation [156], regulates migration in MM [99]
Breast cancer	Involved in tamoxifen resistance [159], required for tumor growth, invasion, and metastasis [22]	Opposes proliferation and tumor growth [22], promotes metastasis [103]
Colorectal cancer	Stable RALA knockdown reduced colony formation via Exo84 [161]	Stable RALB knockdown increased colony formation via SEC5 [161], transient knockdown increased cell death and RALB is upregulated in the CRIS-B subtype [162]
Gastric cancer	Interacts with RCC2 and the MAPK/JNK pathway [163]	Limited data
Hepatic Cancer	Upregulated and activated in cancer tissue [47,66,174], increases migration and stemness [166]	Limited data, does not appear to be upregulated [166]
Pancreatic cancer	Required for tumor establish- ment [176], involved in anchorage-independent growth [84]	Involved in invasion [91]
Lung cancer	Important for tumorigenesis, proliferation, and invasion [167]	Important for invasion [188], cell cycle regulation [56], and migration [169]
Melanoma	Disruption of either RALA or RALB decreases tumor growth [170]	Disruption of either RALA or RALB decreases tumor growth [170]
Nerve sheath tumors	Associated with invasion [171] and apoptosis [172]	Limited data, upstream inhibition of RALB induces apoptosis when NF1 is decreased [172]
Ovarian cancer	GTP-RALA is overexpressed in cancerous cells, important in growth and invasion [173]	Limited data, GTP-RALB is not overexpressed in cancerous cells [173]
Prostate cancer	Central to invasion and migration [93], promotes prostate to bone metastasis [180,181]	Limited data
Renal cancer	Required for invasion [186]	Limited data, not required for invasion [186]

## Data Availability

Not applicable.
